# HMGB1-induced activation of ER stress contributes to pulmonary artery hypertension in vitro and in vivo

**DOI:** 10.1186/s12931-023-02454-x

**Published:** 2023-06-02

**Authors:** Qianqian Zhang, Yuqian Chen, Qingting Wang, Yan Wang, Wei Feng, Limin Chai, Jin Liu, Danyang Li, Huan Chen, Yuanjie Qiu, Nirui Shen, Xiangyu Shi, Xinming Xie, Manxiang Li

**Affiliations:** 1 Department of Respiratory and Critical Care Medicine, The First Affiliated Hospital of Xi’an Jiaotong University, No. 277, West Yanta Road, Xi’an, 710061 Shaanxi China; 2 Department of Respiratory and Critical Care Medicine, The First Affiliated Hospital of Sun Yat-Sen University, Guangzhou, 510080 Guangdong China

**Keywords:** Pulmonary artery hypertension, HMGB1, ER stress, Tetramethylpyrazine

## Abstract

**Background:**

HMGB1 and ER stress have been considered to participate in the progression of pulmonary artery hypertension (PAH). However, the molecular mechanism underlying HMGB1 and ER stress in PAH remains unclear. This study aims to explore whether HMGB1 induces pulmonary artery smooth muscle cells (PASMCs) functions and pulmonary artery remodeling through ER stress activation.

**Methods:**

Primary cultured PASMCs and monocrotaline (MCT)-induced PAH rats were applied in this study. Cell proliferation and migration were determined by CCK-8, EdU and transwell assay. Western blotting was conducted to detect the protein levels of protein kinase RNA-like endoplasmic reticulum kinase (PERK), activating transcription factor-4 (ATF4), seven in absentia homolog 2 (SIAH2) and homeodomain interacting protein kinase 2 (HIPK2). Hemodynamic measurements, immunohistochemistry staining, hematoxylin and eosin staining were used to evaluate the development of PAH. The ultrastructure of ER was observed by transmission electron microscopy.

**Results:**

In primary cultured PASMCs, HMGB1 reduced HIPK2 expression through upregulation of ER stress-related proteins (PERK and ATF4) and subsequently increased SIAH2 expression, which ultimately led to PASMC proliferation and migration. In MCT-induced PAH rats, interfering with HMGB1 by glycyrrhizin, suppression of ER stress by 4-phenylbutyric acid or targeting SIAH2 by vitamin K3 attenuated the development of PAH. Additionally, tetramethylpyrazine (TMP), as a component of traditional Chinese herbal medicine, reversed hemodynamic deterioration and vascular remodeling by targeting PERK/ATF4/SIAH2/HIPK2 axis.

**Conclusions:**

The present study provides a novel insight to understand the pathogenesis of PAH and suggests that targeting HMGB1/PERK/ATF4/SIAH2/HIPK2 cascade might have potential therapeutic value for the prevention and treatment of PAH.

**Supplementary Information:**

The online version contains supplementary material available at 10.1186/s12931-023-02454-x.

## Background

Pulmonary artery hypertension (PAH) is a devastating disease with high mortality and morbidity, hemodynamically characterized by the mean pulmonary arterial pressure (mPAP) > 20 mmHg [1]. Inappropriate vasoconstriction, abnormal pulmonary vascular remodeling and thrombosis in situ are identified as major pathogenesis in PAH [2]. During the development of PAH, the irreversible pulmonary vascular remodeling results from changes of cells in arterial vessel walls, especially pulmonary artery smooth muscle cell (PASMC) excessive proliferation and migration [3]. Current pharmacological therapies for PAH mostly focus on vasomotor pathways, which is inadequate to reach the therapeutic goal for many patients. Novel pathogenic mechanisms and targets of vascular remodeling may provide important insights into PAH treatment.

High-mobility group box 1 (HMGB1), a chromatin-associated protein, stabilizes nucleosomes, thus regulating transcription [4]. Under certain stress conditions, HMGB1 is released from macrophages, monocytes, endothelial cells or various tumor cells. Once released, HMGB1 initiates inflammation and regulates autophagy by binding to toll-like receptor 4 (TLR4) and receptor for advanced glycation end products (RAGE) [5, 6]. Previous studies indicate that HMGB1 and its downstream signaling are involved in PAH pathogenesis [7, 8]. The extracellular or circulating HMGB1 in patients is elevated and used as a biomarker to identify PAH in patients with congenital heart disease [9, 10]. Hypoxia-induced mitogenic factor (HIMF)/HMGB1 signaling axis acts as a pivotal mediator for the proliferation of smooth muscle cells [11]. HMGB1 neutralization or inhibition of TLR4 and RAGE activity represent effective therapeutic strategies for the prevention of PAH [12–14]. However, the specific mechanism by which HMGB1 acts on the progression of PAH is still unclear and needs to be investigated.

Endoplasmic reticulum (ER), as a cellular organelle in eukaryotes, participates in the synthesis, folding, modification and transportation of proteins, involved in the regulation of systemic metabolic, inflammatory, and endocrine processes [15]. Diverse stimuli including hypoxia, nutrient deprivation, aberrant Ca^2+^ regulation and oxidative stress perturb ER homeostasis, leading to accumulation of misfolded and unfolded proteins, and ultimately ER stress [16]. Following ER stress, three sensors of ER homeostasis, inositol-requiring kinase 1 (IRE1), protein kinase RNA-like endoplasmic reticulum kinase (PERK) and activating transcription factor-6(ATF6), are activated to re-establish normal ER function, termed the unfolded protein response (UPR) [17]. Study shows that the PERK-eIF2 signaling cascade is enhanced in the hypoxic bone morphogenetic protein receptor type 2 (BMPR2) heterozygous PASMCs and inhibition of PERK exerts potential antiproliferative effects on PASMCs [18]. All three UPR pathways are activated in the PAH animal models [18–20]. Furthermore, intervention of ER stress by 4-phenylbutyric acid (4-PBA) is beneficial for right ventricular function and prevents the occurrence of PAH [21]. Despite advancement in research on the role of ER stress during PAH, the molecular mechanisms of PERK in PAH are largely undefined.

The mammalian seven-in-absentia homolog 2 (SIAH2) belongs to the RING finger ubiquitin ligase, which is part of a regulatory cascade in the ubiquitin–proteasome system [22]. SIAH2 mediates efficient ubiquitination and degradation of substrates and exerts distinct functions in cellular processes including cell growth, differentiation, angiogenesis and the unfolded protein response [23–25]. Under severe ER stress condition, SIAH2 is an integral component of the ER stress response. ATF4 or IRE1/sXBP1 may constitute the initial signal for SIAH2 transcription, which in turn augments ATF4 availability [23]. In breast cancer cells, SIAH2 partially controls the overall hypoxia response through its effects on the stability of HIF1α, as by ubiquitylation and degradation of homeodomain-interacting protein kinase 2 (HIPK2) [26]. In PAH animal model, SIAH2 promotes pulmonary vascular remodeling through inactivation of YAP [27]. In this study, we assume that HMGB1 triggers ER stress, concomitant with upregulation of SIAH2 and downregulation of HIPK2, leading to PASMC proliferation/migration and pulmonary vascular remodeling.

## Materials and methods

### Cell culture

Primary PASMCs were isolated and cultured from pulmonary arteries of male Sprague–Dawley rats (120–180 g) as previously described [28]. In brief, the main pulmonary arteries were obtained from anesthetized rats. After removing the adventitia and intima carefully, the isolated arteries were shred into small tissue blocks (0.5–1 mm3) and transferred into a culture flask. Then, cells were incubated with high glucose Dulbecco's modified Eagle's medium (DMEM, Gibco Laboratories, Invitrogen, USA) supplemented with 10% fetal bovine serum (FBS, Gemini Bio, Woodland, CA, USA) and 1% penicillin–streptomycin in a humidified incubator at 37 °C aerated with 5% CO2. When cells reached 80% confluency, cells were digested using 0.25% trypsin (Invitrogen, Carlsbad, CA, USA). For maintaining the PASMC phenotype, early-passage cells (passage 3 to 6) were used for all experiments and cell purity was determined by immunostaining for α-smooth muscle actin (α-SMA,1:200) (BM0002, Boster, CA, USA). Cells were starved overnight using a serum-free medium before each experiment. HMGB1 (0-300 ng/ml) (1690-HMB050, R&D systems, Minneapolis, USA) was used to stimulate PASMCs.

### Small interfering RNA (siRNA) transfection

Cells were seeded into 6-well plates for 24 h at approximately 30–40% confluence. Then, cells were transfected with a mixture of Lipofectamine™ 3000 and siRNA. The subsequent experimentations were conducted after transfection for 48 h. The sequences of siRNA duplexes specific for rat PERK, ATF4, SIAH2 and negative control were:

PERK siRNA, sense 5′-GCAGGUCCUUAGUAAUCAUTT-3′, anti-sense 5′-AUGAUUACUAAGGACCUGCTT-3′; ATF4 siRNA, sense 5′-GUCUCUUAGAUGACUAUCUTT-3′, anti-sense 5′-AGAUAGUCAUCUAAGAGACTT′; SIAH2 siRNA: sense 5′-GCAGUUCUGUUUCCCUGUATT-3′, anti-sense 5′-UACAGGGAAACAGAACUGCTT′; negative control (NC) siRNA, sense 5′-UUCUCCGAACGUGUCACGUTT-3′, anti-sense 5′ -ACGUGACACGUUCGGAGAAT-3′. All siRNA was purchased from GenePharma (Shanghai, China).

### Cell proliferation assay

Cell proliferation was determined using cell counting kit-8 (CCK-8) and EdU incorporation assay. Approximately 5 × 10^3^ cells per well were plated into a 96-well culture plate. Three biological replicates of cells were incubated with CCK-8 solution (FD3788, Fudebio-tech, Hangzhou, China) for 2 h. Then, the optical density at 450 nm was measured using a microplate reader. For the EdU incorporation assay, cells were labeled with EdU (C0071S, Beyotime, Shanghai, China) for 4 h at 37 °C. The positive cells were observed under inverted fluorescence microscopy and calculated using Image J software (NIH, Bethesda, MD, USA).

### Cell migration assay

After different treatments, cells (5 × 10^4^ cells/well) in the serum-free medium were collected and seeded into the upper chamber of 24-well transwell chambers (Corning Inc, USA). The lower chamber was filled with 500 µl DMEM containing 10% FBS with or without HMGB1. Then, cells traversed the membrane were fixed with 4% (w/v) paraformaldehyde for 20 min and stained with 0.1% crystal violet for 10 min at room temperature. The number of migrated cells was counted under an inverted microscope.

### Animal experiment

Male Sprague Dawley rats were purchased from Xi'an Jiaotong University Experimental Animal Center. All procedures involved in the experiment were approved by the Institutional Animal Ethics Committee of Xi'an Jiaotong University and under the Guide for the Care and Use of Laboratory Animals of Xi'an Jiaotong University Animal Experiment Center. Rats were kept in a temperature-controlled room (20 ± 2 °C) with a 12 h light/dark cycle and maintained on a standard diet. In this study, all rats weigh approximately 200–220 g. PAH rats were induced by a single intraperitoneal(ip) injection of 60 mg/kg MCT (Must Bio-Technology, Chengdu, China) on day 1, while control animals (n = 8) were administered 0.9% NaCl solution. Then MCT-injected rats were randomly divided into six groups (n = 8 per group) as follows: MCT group; MCT + DMSO group: received DMSO vehicle; MCT + Glycyrrhizin (GLY) group: received GLY (100 mg/kg, 53,956–04-0, Santa Cruz, CA, USA) by daily ip injection; MCT + 4-phenylbutyric acid (4-PBA) group: received 4-PBA (500 mg/kg, HY-A0281, MedChemExpress, Monmouth Junction, America) by daily gavage; MCT + Vitamin K3(VK3) group: received VK3 (3.5 mg/kg, HY-B0332, MedChemExpress, Monmouth Junction, America) by ip injection twice a week; MCT + Tetramethylpyrazine(TMP) group: received TMP(100 mg/kg, Lizhu Pharmaceutical Limited Company, Zhuhai, China) by daily gavage.

### Hemodynamic measurements

For measurement of hemodynamic parameters, rats were anesthetized using 2% pentobarbital sodium (0.3 ml/100 g). A catheter was inserted into the right pulmonary artery through the right external jugular vein and then the right ventricle by closed-chest technique. Right ventricular systolic pressure (RVSP) and mPAP were assessed carefully. After that, we dissected the right ventricle (RV) and left ventricle (LV) plus interventricular septum (S). The right ventricular hypertrophy was assessed by the RV/LV + S ratio.

### Histology, immunohistochemistry staining and double-labeling immunofluorescence staining

Lung and heart specimens were fixed in 4% formalin, embedded in paraffin and sectioned longitudinally at a thickness of 5 µm. Slides were stained with hematoxylin–eosin (HE) and Elastic van Gieson (EVG) using previous protocols [29]. The percentage of medial wall thickness was measured n distal pulmonary arteries (20–70 μm diameters, n = 30 per rat). Images were captured using a light microscope (CellSens Imaging Software, Olympus, Tokyo, Japan). For immunohistochemistry staining, paraffin-embedded lung sections were incubated with α-SMA (#14395-1-AP, Proteintech, Wuhan, China) overnight at 4 °C. Semi-quantitative analysis for staining of α-SMA was conducted to categorize the degree of pulmonary arterial muscularization. The co-staining of α-SMA and ATF4 was conducted to determine the expression of ATF4 in the PASMCs. Lung sections were incubated with α-SMA (1:50 dilution) and ATF4 (1:100 dilution) at 4 °C overnight. Then, sections were incubated with the fluorescent secondary antibody (1:250) and DAPI. Afterward, sections were observed and photographed by an inverted fluorescence microscope (Leika Microsystems, Wetzlar, Germany).

### Transmission electron microscopy

The left lower lobe of the lung was removed from rats and fixed in 2.5% (w/v) glutaraldehyde. Pulmonary arteries were isolated from lungs and postfixed with 1% (w/v) OsO4, dehydrated by alcohol and then embedded in araldite. Ultrathin sections were sliced from the specimens and mounted on copper grids. Then, sections were stained with 2% uranyl acetate and lead citrate. A transmission electron microscope (TEM) (H-7650, Hitachi, Japan) was used to observe and evaluate the ultrastructure of ER.

### Western blotting

The lung tissues of rats were cut into pieces and homogenized using cold RIPA buffer (Beyotime, Shanghai, China). Total protein was obtained from the lysed tissue homogenate centrifuged at 10,000 rpm at 4 °C for 20 min. Total cellular proteins were also extracted using the RIPA lysis buffer. All protein concentrations were quantified with the bicinchoninic acid kit (Beyotime Shanghai, China). Then, proteins from each sample were separated by 10% SDS-PAGE and electro-blotted onto polyvinylidene fluoride membranes (Millipore, Billerica, MA). The membranes were blocked using 5% non-fat milk for 60 min at room and incubated with primary antibodies overnight at 4 °C while shaking. Rabbit monoclonal antibodies against PERK (#3192) and ATF4 (#11815) were purchased from Cell Signaling Technology (Beverly, MA, USA). Rabbit polyclonal antibody against SIAH2 (#YT4297) and mouse monoclonal antibody against β-actin (#YM3028) were from Immunoway (Plano, TX, USA). Rabbit monoclonal antibody against HIPK2 (#ab108543) was from Abcam (Boston, MA, USA). To detect the primary antibody, the membranes were incubated with a horseradish peroxidase-conjugated anti-rabbit or anti-mouse IgG antibody diluted 1:5000 to 1:10,000 for 1 h at room temperature. Chemiluminescence was performed using the ChemiDoc XRS system and analyzed with ImageJ software.

### Statistical analysis

Results were presented as mean ± standard deviation (SD). All experiments were conducted for at least three independent replications. The data were applied to Shapiro–Wilk normality test and F test for normality and equal variance tests, respectively. The student’s t-test determined statistical differences between two groups. For comparisons within multiple groups, one-way ANOVA followed by Tukey’s multiple comparisons post-hoc test was used. Statistical analyses were performed using GraphPad Prism version 8.0 (GraphPad Software, La Jolla, CA, USA). The significant difference was assumed at P-value < 0.05.

## Results

### HMGB1 induces ER stress-associated proteins (PERK and ATF4) and SIAH2 upregulation, HIPK2 downregulation and PASMC proliferation and migration

**Fig. 1:**
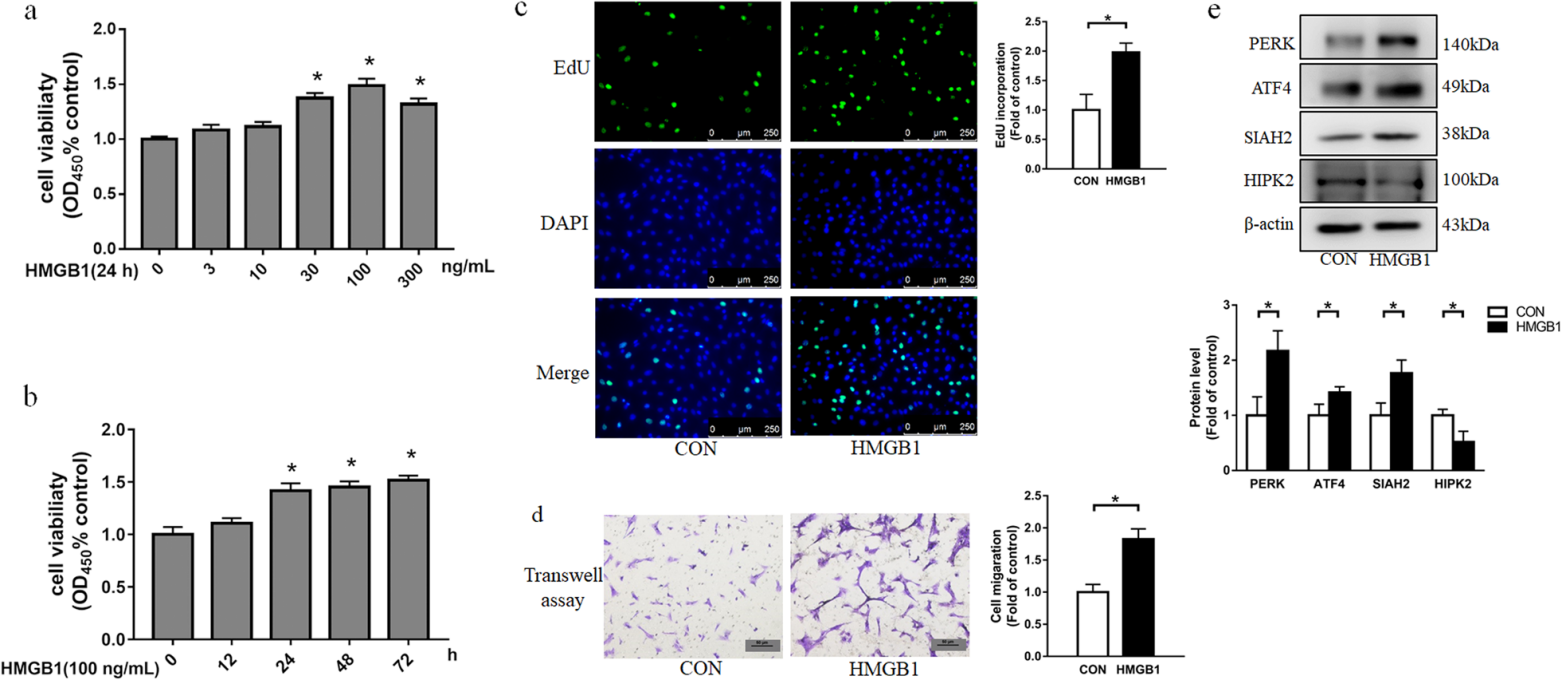
HMGB1 promotes PASMC proliferation/migration, and induces ER stress activation, SIAH2 upregulation and HIPK2 downregulation. **a** Cells were incubated with HMGB1 for 24 h at concentrations ranging from 0 to 300 ng/ml. Cell viability was evaluated using the Cell Counting Kit-8(CCK-8) assay (n = 6 per group).** b** Cells were stimulated with 100 ng/ml HMGB1 for different times (0, 12, 24, 48, 72 h). Cell viability was evaluated using the Cell Counting Kit-8(CCK-8) assay (n = 6 per group). PASMCs were exposed to 100 ng/ml HMGB1 for 24 h. Cell proliferation was measured by EdU incorporation assay (scale bar = 250 μm) **(c)**; Cell migration was measured by transwell assay (scale bar = 50 μm)** (d)**; PERK, ATF4, SIAH2 and HIPK2 expression were measured using western blotting** (e)**. For original blot images, see Additional file 1. *P < 0.05

In PAH, the initial damage of pulmonary vascular cells induces HMGB1 release and increases the level of circulated HMGB1, which is involved in the severe phenotype of PAH [30]. To evaluate the contribution of HMGB1 in PASMC proliferation and migration, cells were treated with HMGB1 ranging from 0 to 300 ng/ml for 24 h or at 100 ng/ml for different time (0, 12, 24, 48 and 72 h). As shown in Fig. 1a and b, HMGB1 stimulated PASMC proliferation dose- and time-dependently. 100 ng/ml HMGB1 caused the most obvious increase in cell viability, which was used in subsequent cell experiments. Results of the EdU assay also showed that the number of positive cells in HMGB1group was increased by 1.98-fold at 24 h compared with control (Fig. 1c). Cell migration was detected by transwell assay and the results indicated that the number of migrating cells was increased by 1.83-fold in PASMCs treated with 100 ng/ml HMGB1 for 24 h (Fig. 1d). These results indicate that HMGB1 induces PASMC proliferation and migration. To investigate the mechanisms underlying HMGB1-induced PASMC proliferation and migration, we detected ER stress-associated proteins (PERK and ATF4), SIAH2 and HIPK2 expression. As shown in Fig. 1e, PERK, ATF4 and SIAH2 expression were increased, and HIPK2 was decreased in PASMCs stimulated with HMGB1 for 24 h. Thus, we performed subsequent experiments based on these findings.

### PERK/ATF4 mediates HMGB1-induced SIAH2 upregulation and HIPK2 downregulation

**Fig. 2:**
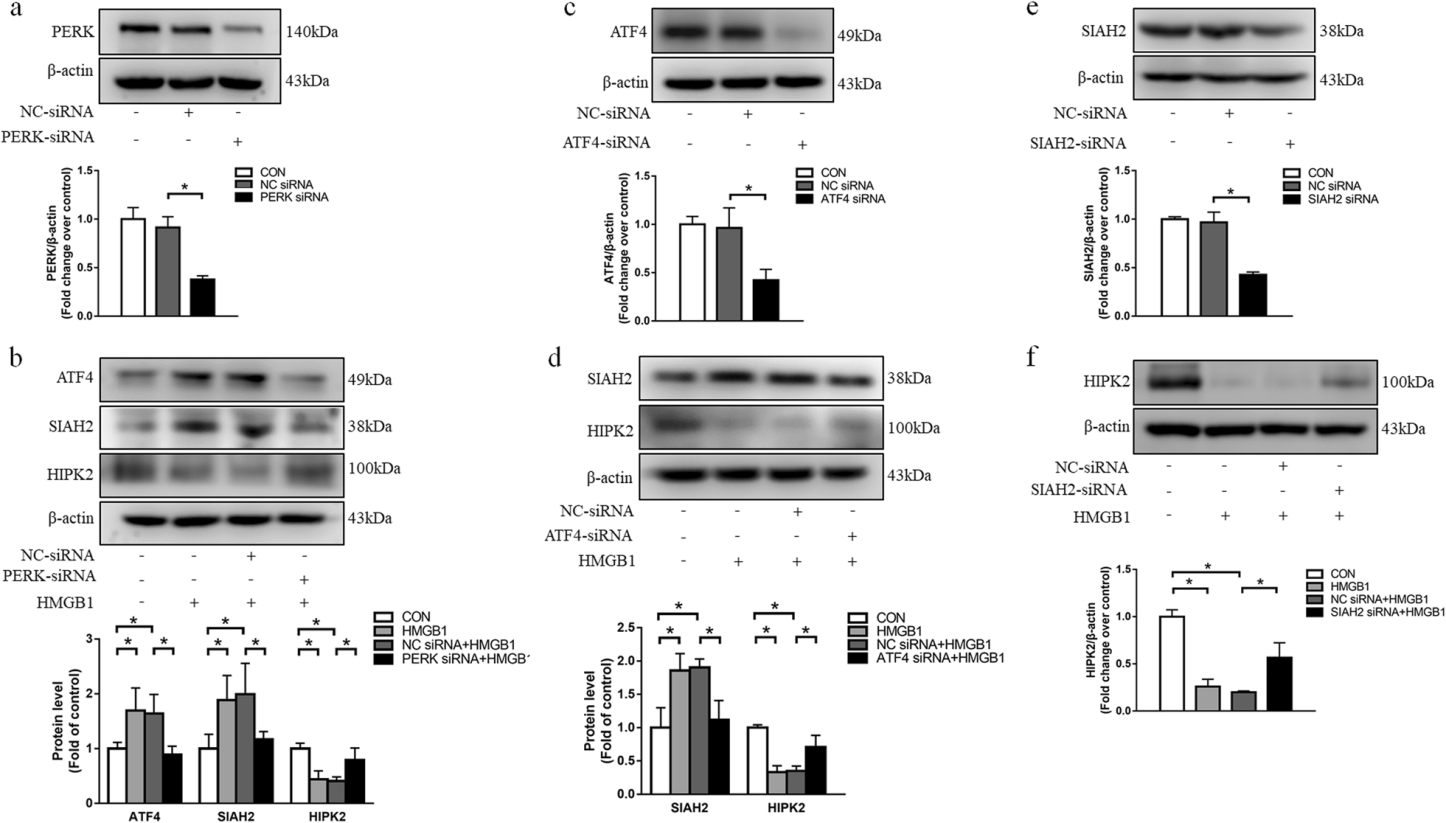
ER stress mediates HMGB1-induced SIAH2 upregulation and HIPK2 downregulation. The silencing effects of PERK **(a)**, ATF4 **(c)** and SIAH2** (e)** were evaluated by western blotting after specific siRNA or nontargeting siRNA transfection in cells for 48 h, respectively. **b, d, f** Specific siRNA or nontargeting siRNA was transfected into cells for 24 h, then cells were exposed to 100 ng/ml HMGB1for 24 h. ATF4, SIAH2 and HIPK2 protein levels were measured using western blotting. For original blot images, see Additional file 1. *P < 0.05

It has been reported that PERK/ATF4 induces transcription and expression of the ubiquitin ligases SIAH1/2 in cancer cell lines[23].To identify the link among PERK, ATF4, SIAH2 and HIPK2, knockdown of genes by siRNA was carried out. Transfection efficiency of PERK, ATF4 and SIAH2-siRNA were shown in Fig. 2a, c and e, respectively. Figure 2b and d demonstrate that HMGB1-induced upregulation of ATF4 was declined in cells prior transfected with PERK-siRNA. Silencing of PERK or ATF4 reduced HMGB1-induced upregulation of SIAH2 and increased HMGB1-caused HIPK2 downregulation. These results suggest that PERK/ATF4 pathway acts upstream of SIAH2 and HIPK2 in PASMCs.

We further explored the effect of loss of SIAH2 on HIPK2 expression. As shown in Fig. 2f, SIAH2 knockdown reversed HMGB1-induced HIPK2 downregulation, suggesting that SIAH2 is one of the important negative regulators of HIPK2 expression in PASMCs.

### ER stress and SIAH2 mediate HMGB1-induced proliferation and migration in PASMCs

**Fig. 3:**
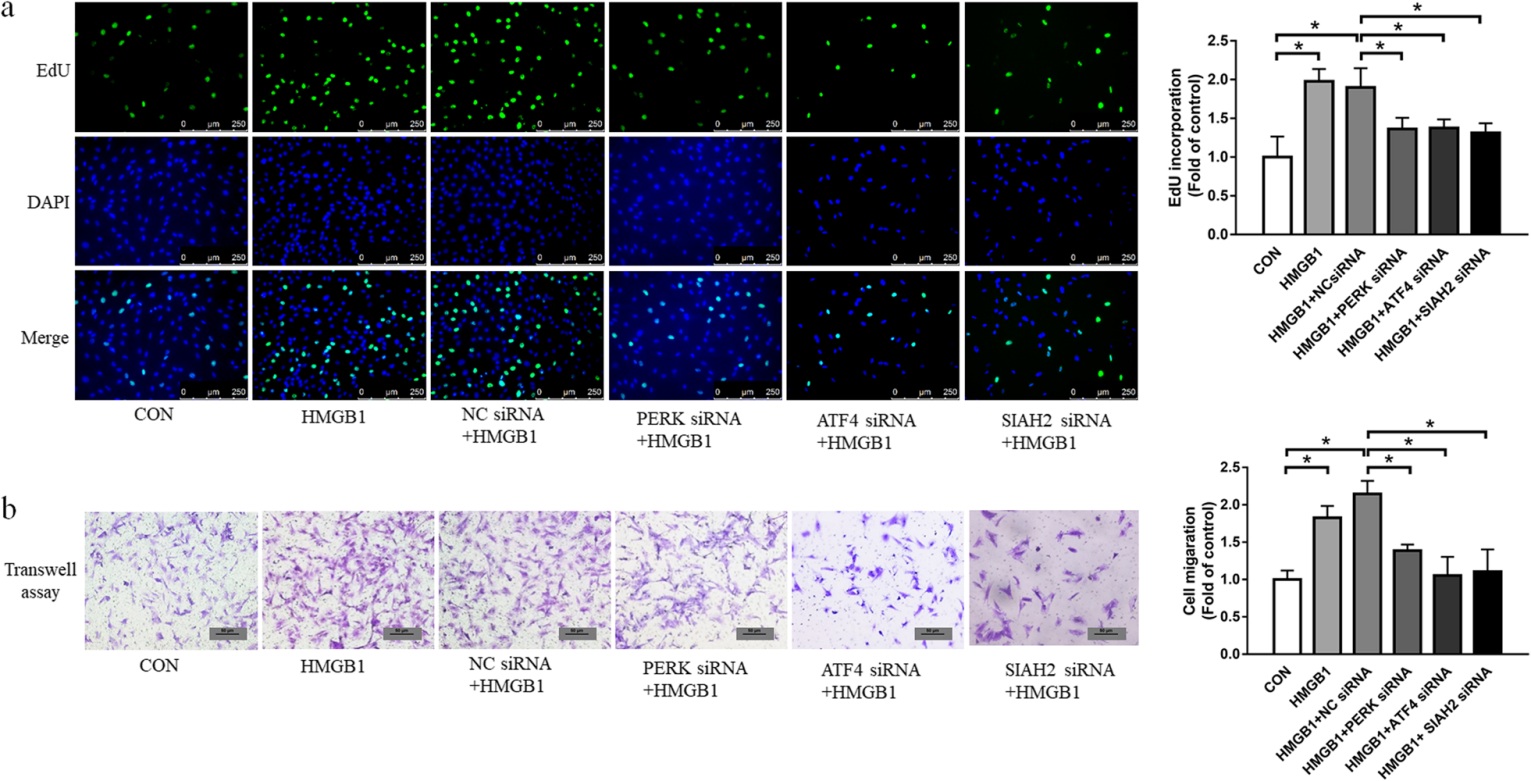
HMGB1 stimulates PASMC proliferation and migration through PERK/ATF4/SIAH2/HIPK2 pathway. PASMCs were transferred with PERK siRNA, ATF4 siRNA, SIAH2 siRNA or NC siRNA for 24 h, then incubated with 100 ng/ml HMGB1 for 24 h. Cell proliferation was detected by EdU incorporation assay** (a)** and cell migration was measured by transwell assay **(b)**. *P < 0.05

We further investigate whether ER stress and SIAH2 participated in cellular functions of PASMCs. To elucidate the influence of this pathway on cell proliferation, cells were transfected with NC-siRNA, PERK-siRNA, ATF4-siRNA or SIAH2-siRNA for 24 h before stimulation with HMGB1 for 24 h. As shown in Fig. 3a, HMGB1-induced cell proliferation was significantly inhibited by knockdown of PERK, ATF4 or SIAH2, measured by the EdU assay. Next, we evaluated the capability of cell migration in PASMCs under these conditions using the transwell assay. Figure 3b indicates that HMGB1 stimulation resulted in an obvious increase in the number of migrating cells, which was suppressed by silencing PERK, ATF4 or SIAH2. Collectively, these functional studies suggest that ER stress and SIAH2 mediate PASMC proliferation and migration triggered by HMGB1.

### TMP suppresses HMGB1-induced proliferation and migration of PASMCs via PERK/ATF4/SIAH2/HIPK2 axis

**Fig. 4:**
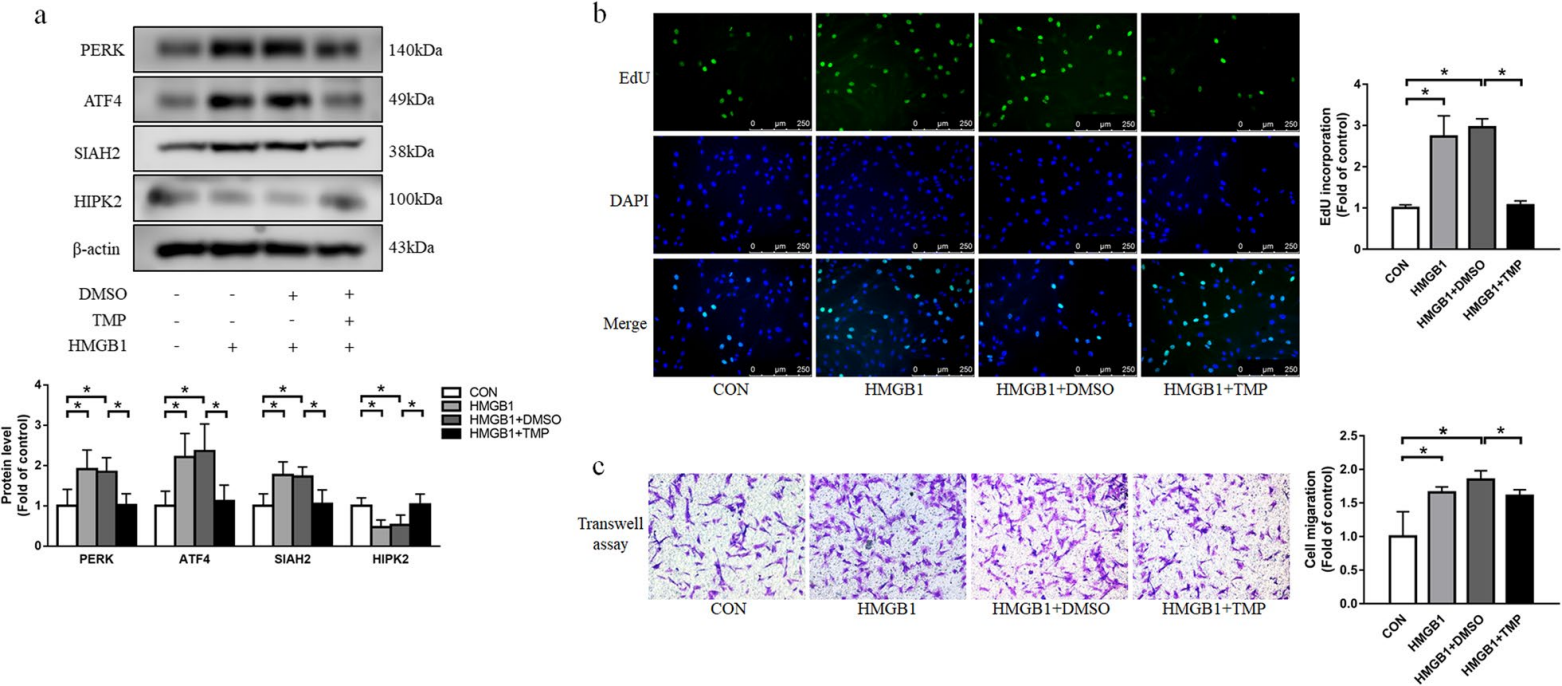
TMP inhibits HMGB1-induced PASMC proliferation and migration via PERK/ATF4/SIAH2/HIPK2 axis. Cells were incubated with HMGB1 and TMP for 24 h. **a** PERK, ATF4, SIAH2 and HIPK2 expression were measured using western blotting. **b** Cell proliferation was determined by EdU incorporation assay. **c** Cell migration was detected using transwell assay. For original blot images, see Additional file 1. *P < 0.05

Tetramethylpyrazine (TMP) is a compound isolated from the traditional Chinese herb ligusticum, which has diverse functions including anti-oxidation, anti-platelet aggregation and anti-inflammation [31]. It has been reported that TMP has potent effects for the treatment of pulmonary hypertension by scavenging intracellular ROS[32; 33].To clarify the effect of TMP in PASMCs, cells were incubated with TMP for 24 h in the presence of HMGB1. As shown in Fig. 4a, TMP administration inhibited HMGB1-induced PERK, ATF4 and SIAH2 upregulations, and HIPK2 downregulation. Furthermore, we found that HMGB1-induced elevations of PASMC proliferation and migration were also suppressed by TMP treatment (Fig. 4b, c). Collectively, these findings demonstrate that TMP negatively regulates PERK/ATF4/SIAH2 axis to prevent excessive PASMC proliferation and migration.

### Inhibition of HMGB1 attenuates vascular remodeling in the MCT-induced PAH model

**Fig. 5:**
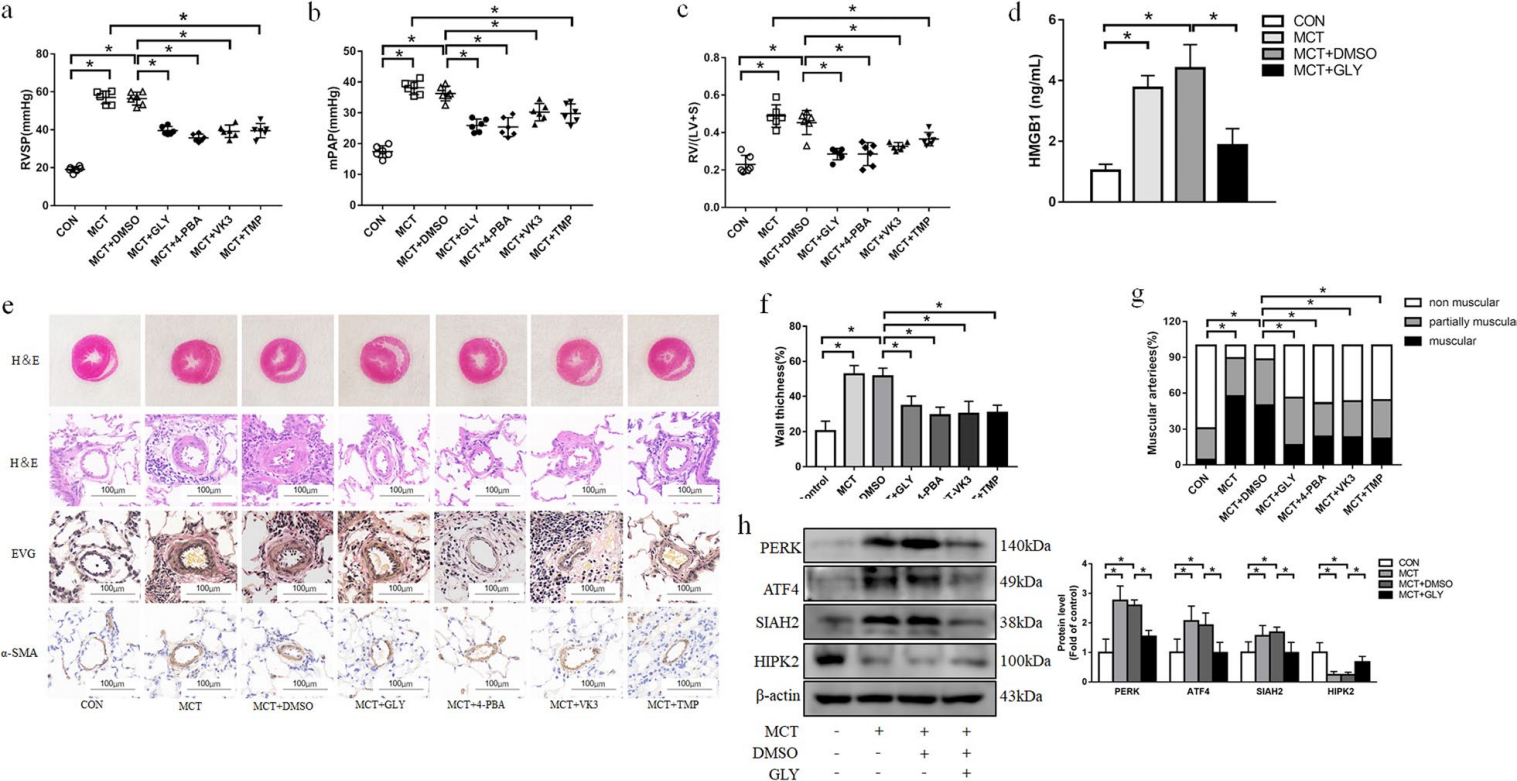
Inhibition of HMGB1, ER stress or SIAH2 reverse pulmonary hemodynamic parameters and vascular remodeling. Comparison of RVSP **(a)**, mPAP **(b)**, the ratio of RV/(LV + S)** (c)** in each group. **d** the HMGB1 serum level in different groups was detected by ELISA. **e** Representative images of hematoxylin and eosin (HE) staining revealed RV hypertrophy; HE staining and Eastic Van Gieson (EVG) staining of distal pulmonary arterioles reflect the medial wall thickness of pulmonary; Immunohistochemical staining of α-SMA revealed the muscularization of distal pulmonary arterioles. scale bar = 100 μm. **f** Quantitative analysis of the percentage of the medial wall thickness of pulmonary arteries. **g** Quantitative analysis of muscularization of distal pulmonary arteries. RVSP: right ventricle systolic pressure; mPAP mean pulmonary arterial pressure; RV/(LV + S): ratio of the right ventricle to left ventricle plus septum. **h** Protein levels of PERK, ATF4, SIAH2 and HIPK2 in lung tissues from each group were measured by immunoblotting. For original blot images, see Additional file 1. *P < 0.05

Based on our studies of cells, we hypothesized that HMGB1 might be involved in the PAH model via PERK/ATF4/SIAH2/HIPK2 axis mediated vascular remodeling. To address this issue, the rat model of PAH was induced successfully by intraperitoneal injection of MCT (60 mg/kg), which was manifested as remarkable elevations of RASP (Fig. 5a), mPAP (Fig. 5b) and RV/(LV + S) (Fig. 5c). We next detected the HMGB1 concentrations in serum and found that the serum HMGB1 level was increased in the MCT group compared with control, whereas a significant decline was observed in the MCT group treated with GLY (HMGB1 inhibitor) compared with the MCT group (Fig. 5d). In addition, treatment with GLY also reduced RASP (Fig. 5a), mPAP (Fig. 5b) and RV/(LV + S) (Fig. 5c) elevation in PAH model. To evaluate the distal pulmonary vascular abnormalities, we conducted histological studies. As shown in Fig. 5e–g, right ventricular hypertrophy, medial wall thickness and muscularization of pulmonary arteries were elevated in the MCT-induced PAH model. However, GLY administration alleviated all these changes. These results suggest that inhibition of HMGB1 improves hemodynamic deterioration and vascular remodeling in the MCT-induced PAH rats.

We further investigated the effects of HMGB1 inhibition on the PERK/ATF4/SIAH2/HIPK2 cascade. As shown in Fig. 5h, PAH rats exhibited up-regulation of PERK, ATF4 and SIAH2 and down-regulation of HIPK2, while treatment of PAH rats with GLY reversed these changes.

### Suppression of ER stress alleviates pulmonary vascular remodeling by SIAH2 downregulation and HIPK2 upregulation

**Fig. 6:**
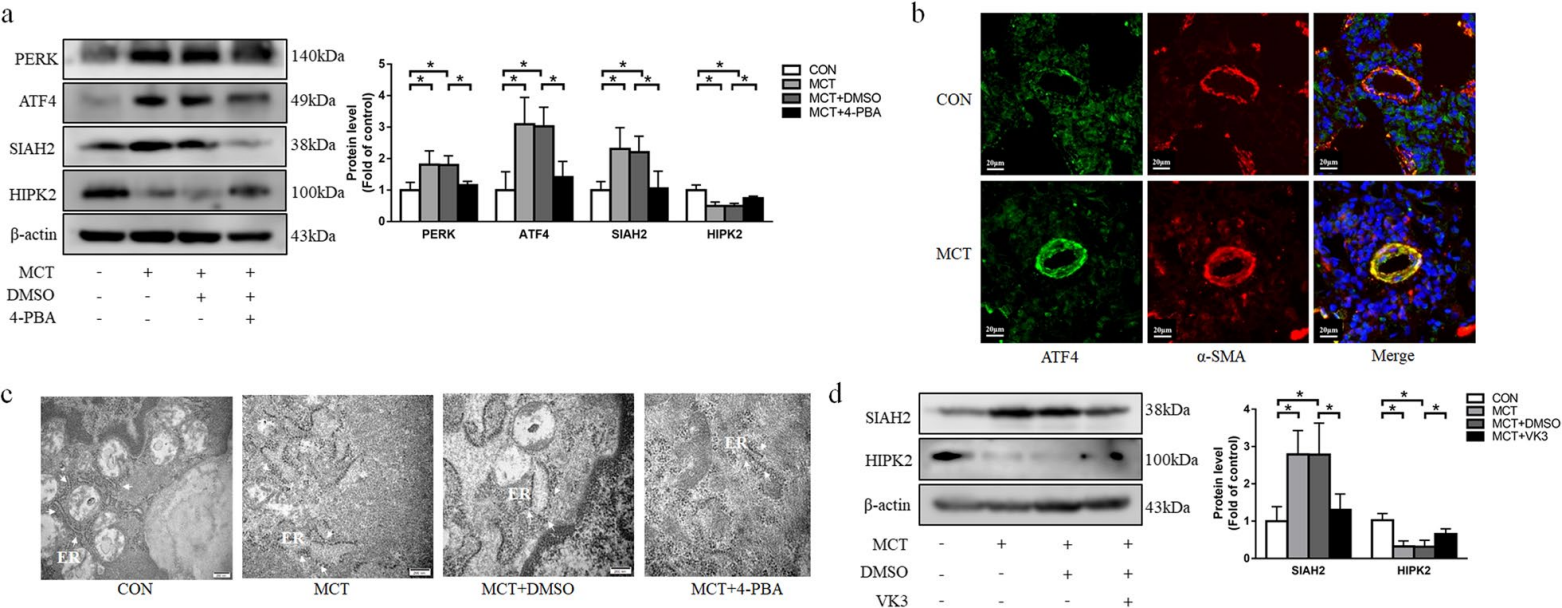
Suppression of ER stress activation or SIAH2 induces HIPK2 expression. **a** protein levels of PERK, ATF4, SIAH2 and HIPK2 in lung tissues from each group were measured by immunoblotting. **b** Immunofluorescence of ATF4 in pulmonary arteries. ATF4 (green), α-SMA (red) and nucleus (blue) were stained in lung tissue from different groups. **c** Representative images of ER morphology by transmission electron microscopy. Scale bar = 200 nm. ER, endoplasmic reticulum. **d** Protein levels of SIAH2 and HIPK2 were determined by immunoblotting. For original blot images, see Additional file 1. *P < 0.05

It has been reported ER stress acts as an important cellular response in the pathogenesis of pulmonary artery hypertension [34]. To determine whether ER stress mediates pulmonary vascular remodeling in the MCT-induced PAH model, the ER stress indicators were measured by western blotting. As shown in Fig. 6a, the expression of PERK and ATF4 was increased in the MCT group. Immunofluorescence staining also revealed an increased expression of ATF4 in the distal pulmonary arteries of PAH rats (Fig. 6b). We further assessed the ultrastructure of PASMC using transmission electron microscopy. The swollen ER with remarkable expansion of the intracisternal space was observed in the PAH rats (Fig. 6c). These data indicate that ER stress is induced in the PAH rats.

We next explored whether inhibition of ER stress has a beneficial effect on the progression of PAH in vivo. A molecular chaperone, 4-PBA, was used to prevent ER stress. As shown in Fig. 6a, elevated protein levels of PERK and ATF4 in the PAH rat declined after 4-PBA treatment. The swollen ER with disruption of luminal structures was ameliorated in 4-PBA treated PAH rats (Fig. 6c). 4-PBA treatment also alleviated the hemodynamic changes in pulmonary arteries and right ventricular structure including RASP (Fig. 5a), mPAP (Fig. 5b) and RV/(LV + S) (Fig. 5c). Lung histological analysis revealed that right ventricular hypertrophy, pulmonary arterioles wallthickness and muscularizedarteries were ameliorated after 4-PBA treatment (Fig. 5e–g).

To determine whether SIAH2 and HIPK2 mediate the effect of ER stress on MCT-induced PAH rats, we examined the expression of SIAH2 and HIPK2. As shown in Fig. 6a, MCT increased the SIAH2 protein level and reduced the HIPK2 protein level. However, 4-PBA treatment reversed the changes of SIAH2 and HIPK2 expression in PAH rats. In addition, Fig. 6d indicates that VK3, a SIAH2 inhibitor, decreased SIAH2 up-regulation and blocked HIPK2 down-regulation in PAH rats. VK3 administration also reduced the elevation of RASP, mPAP and RV/(LV + S) (Fig. 5a–c) and ameliorated the right ventricular hypertrophy, pulmonary arterioles wall thickness and muscularization of distal pulmonary arteries (Fig. 5e–g) compared with the MCT group. Taken together, these results demonstrate that inhibition of ER stress alleviates pulmonary vascular remodeling and the development of PAH through SIAH2/HIPK2 pathway.

### TMP inhibits ER stress and prevents the development of PAH in rat

**Fig. 7:**
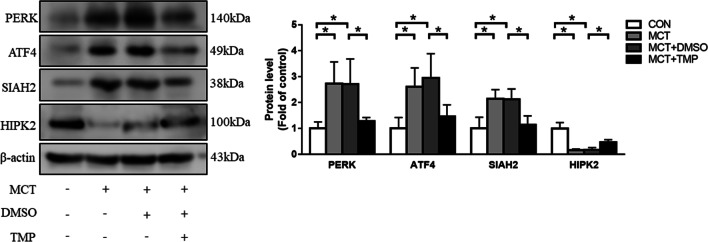
The underlying mechanism of TMP protective effect against MCT-induced PAH. PERK, ATF4, SIAH2 and HIPK2 protein levels in lung tissue were determined from each group using immunoblotting. For original blot images, see Additional file 1. * *P* < 0.05

TMP treatment significantly improves 6-min walking distance and right heart function in both the prevention and treatment of PAH model [35]. In coronary arteries, TMP improves BK_Ca_-mediated vasodilation by suppression of ER stress [36].To explore whether TMP prevents MCT-induced the development of PAH in rats by targeting ER stress, TMP was applied to rats after MCT injection. Figure 7 shows that TMP administration reduced MCT-induced elevation of PERK, ATF4 and SIAH2 protein expression and raised HIPK2 expression.

We further assessed the hemodynamic and histologic changes. The results showed that TMP reduced RASP, mPAP and RV/(LV + S) elevation in PAH rats (Fig. 5a–c). Histological examination indicated that right ventricular hypertrophy, pulmonary arterioles wall thickness and pulmonary arteries muscularization induced by MCT were relieved after TMP treatment (Fig. 5e–g). Collectively, these data suggest that TMP treatment effectively attenuates the progression of PAH in rats.

## Discussion

The biological functions of HMGB1 are dependent on its diverse cellular localization. When response to persistent tissue injury, HMGB1 is released into the extracellular environment as a key molecule of innate immunity, inflammation and tissue remodeling [37]. In IPAH patients, extranuclear HMGB1 is observed in plexiform vascular lesions. Circulating HMGB1 levels increase and correlate with the severity of PAH [38]. Our study investigated the role of extracellular HMGB1 in PASMCs and the pathogenesis of MCT-induced PAH rats. We found that HMGB1 promoted PASMC proliferation and migration. In vivo, the HMGB1 serum level was elevated, whereas inhibition of HMGB1 by GLY reduced the HMGB1 concentration in the serum and improved pulmonary hemodynamics and vascular remodeling.

Accumulated evidence indicates that ER stress participates in diverse PAH-triggering and PAH-facilitating processes such as inflammation, hypoxia and genetic mutation [34, 39]. All branches of the UPR under ER stress are activated, accompanied by inflammatory responses in chronic hypoxia-induced PAH. In lung sections from IPAH patients, unfolded protein response triggered by ER stress is evident [40]. Moreover, PERK mediates the C/EBP-homologous protein (CHOP) transcriptional activation and participates in hypoxia-induced dysfunction of HPAECs [41]. In this study, we found that PERK/ATF4 expression was up-regulated by HMGB1 and inhibition of PERK/ATF4 suppressed proliferation and migration of PASMCs. 4-PBA, a chemical chaperone, has been identified as an inhibitor of ER stress[42; 43] and has reduced the expression of ER stress indicators, including GRP78, ATF6, IRE-1 and PERK [21; 44]. We showed that ER stress was obvious in the MCT-induced PAH rat model, indicated by the morphological change of ER and elevation of PERK/ATF4 expression. Moreover, 4-PBA application inhibited PERK/ATF4 expression and contributed to the reversal of pulmonary artery vascular remodeling.

SIAH2 is a member of the seven in absentia homolog family proteins, comprising a C-terminal substrate-binding domain, a catalytic RING domain, and two zinc fingers [45]. SIAH2 is involved in different fundamental cellular processes and activated by various stress conditions and intracellular signaling pathways [46]. It has been reported that ER stress induces the transcription of SIAH2 [23]. In the present study, we found that HMGB1 promoted SIAH2 expression by PERK/ATF4 axis in PASMCs and PAH rats. Furthermore, VitaminK3 as a novel inhibitor suppressed SIAH2 expression, inhibited PASMC proliferation and migration, and ultimately reversed vascular remodeling in PAH.

HIPK2 is a conserved serine/threonine kinase that modulates several biological responses, including cell proliferation, apoptosis, and DNA damage response [47, 48]. As a signal transduction element, HIPK2 regulates molecular pathways that contribute to diabetes, nephropathy, idiopathic pulmonary fibrosis, cardiac disease and several cancers [49–53]. HIPK2 overexpression plays a crucial role in promoting apoptosis in diverse cell types [54, 55]. In hepatocellular carcinoma, HMGBI promotes ubiquitination and degeneration of HIPK2, which results in autophagy induction and tumor progression [56]. In myocardial infarction, exercise reduces HIPK2 protein level, leading to the prevention of cardiomyocytes apoptosis and elevation of cardiac function [57]. Consistent with these studies, we observed that HIPK2 expression was down-regulated in HMGB1-treated PASMCs and in MCT-induced PAH rats through PERK/ATF4/SIAH2 pathway.

TMP, an amide alkaloid, is the main bioactive active component of a traditional Chinese herbal medicine, Chuanxiong [58]. TMP exerts potent effects in anti-cancer, anti-oxidation, anti-inflammation and antithrombotic [59, 60]. At present, TMP is widely used in the clinic for the treatment of cardiovascular [61], cerebral ischemia [62], cancer [63] and pulmonary hypertension [33]. The curative effects of TMP have been shown in PAH patients indicated by the increase of average 6-min walk distance and right heart function. In PAH rats, hypoxia is an important trigger for the increase in [Ca^2+^]. TMP inhibits the intracellular Ca^2+^ signaling in PASMCs and reverses established PH in rats [35]. Several studies also show that TMP exerts protective effects on various diseases via inhibition of ER stress [64–66]. In coronary endothelial cells, TMP prevents Ang-II-induced endothelial dysfunction by blocking the phosphorylation of PERK and upregulation of ATF4 [65]. In the present study, we found that TMP treatment suppressed activation of ER stress, decreased SIAH2 expression and increased HIPK2 expression, ultimately prevented PASMC proliferation/migration and ameliorated pulmonary vascular remodeling in MCT-induced PAH rats. These results are consistent with the previous study and indicate that PERK/ATF4/SIAH2/HIPK2 might be the molecular mechanism of TMP to maintain the function of pulmonary artery vascular and to inhibit the development of PAH.

## Conclusion

In the present study, our study evaluated the crucial role of ER stress in the development of PAH. First, we observed that HMGB1 induced activation of ER stress, upregulation of SIAH2 and downregulation of HIPK2 in PASMCs and MCT-induced PAH rat model. Furthermore, GLY, 4-PBA and VK3 administration attenuated the increases of RVSP, mPAP and RV/(LV+S), right ventricular hypertrophy, and pulmonary vascular remodeling by targeting on PERK/ATF4/SIAH2/HIPK2 pathway in PAH rats. Our results also demonstrated that TMP as a traditional Chinese medicine inhibited PASMCs proliferation and migration, and blocked the progression of PAH through inhibition of ER stress in PAH model. Based on the history of safe usage and high efficacy of TMP, it might be an ideal and potential drug for the treatment of PAH.

## Supplementary Information

**Additional file 1**. The original blot images for Figs. 1, 2, 4, 5, 6 and 7. Expression of PERK, ATF4, SIAH2 and HIPK2 were determined from each group using immunoblotting.

## Data Availability

The data that support the findings of this study are available from the corresponding author upon reasonable request.

## Abbreviations

HMGB1: High-mobility group box 1
ER: Endoplasmic reticulum
PAH: Pulmonary artery hypertension
PASMCs: Pulmonary arterial smooth muscle cells
MCT: Monocrotaline
CCK-8: Cell Counting Kit-8
HIPK2: Homeodomain interacting protein kinase 2
SIAH2: Seven in absentia homolog 2
PERK: Protein kinase RNA-like endoplasmic reticulum kinase
ATF4: Activating transcription factor-4
GLY: Glycyrrhizin
4-PBA: 4-Phenylbutyric acid
VK3: Vitamin K3
TMP: Tetramethylpyrazine
mPAP: Mean pulmonary arterial pressure
TLR4: Toll-like receptor 4
RAGE: Advanced glycation end products
IRE1: Inositol-requiring kinase 1
ATF6: Activating transcription factor-6
UPR: Unfolded protein response
α-SMA: α-Smooth muscle actin
siRNA: Small interfering RNA
CCK-8: Cell counting kit-8
DMEM: Dulbecco’s modified eagle medium
FBS: Fetal bovine serum
HE: Hematoxylin–eosin
EVG: Elastic van Gieson
RVSP: Right ventricular systolic pressure
TEM: Transmission electron microscope
SD: Standard deviation
