# Supplementary material for: HMGB1-induced activation of ER stress contributes to pulmonary artery hypertension in vitro and in vivo
Source: Respir Res. 2023 Jun 2;24:149. doi: 10.1186/s12931-023-02454-x (PMC10236651; doi:10.1186/s12931-023-02454-x)

Figure1:

PERK(1)

PERK(2)

PERK(3)

PERK(4)

ATF4 (1)

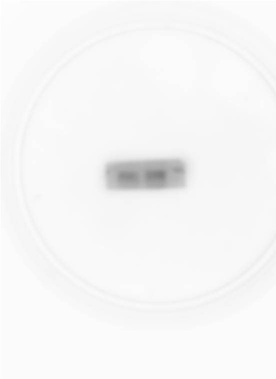

ATF4 (2)

ATF4 (3)

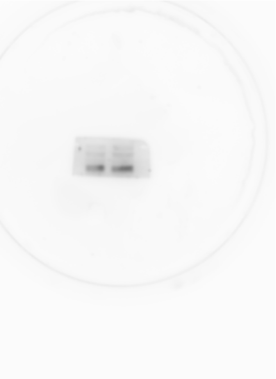

ATF4 (4)

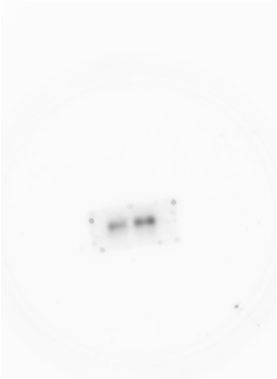

SIAH2(1)

SIAH2(2)

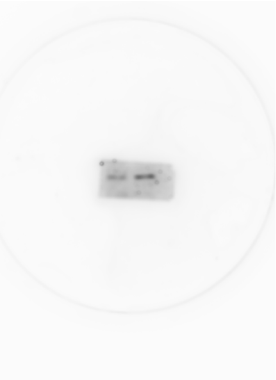

SIAH2(3)

SIAH2(4)

HIPK2(1)

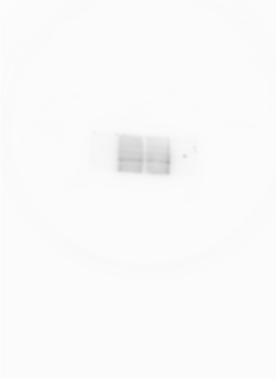

HIPK2(2)

HIPK2(3)

HIPK2(4)

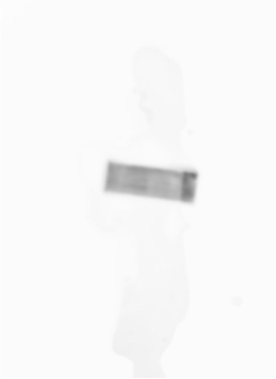

β -actin(1)

β -actin(2)

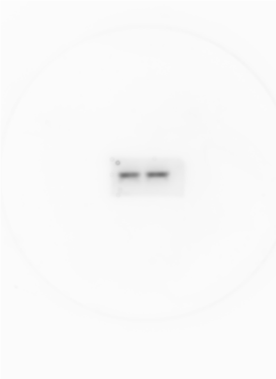

β -actin(3)

β -actin(4)

Figure2a:

PERK(1) :
the first brand

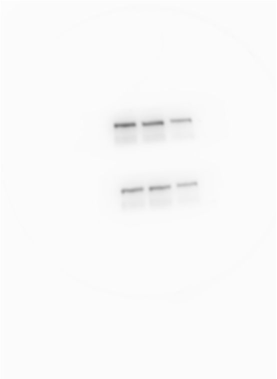

PERK(2):
the first brand

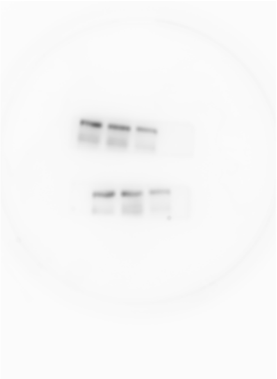

PERK(3) and (4):
the second and third brands

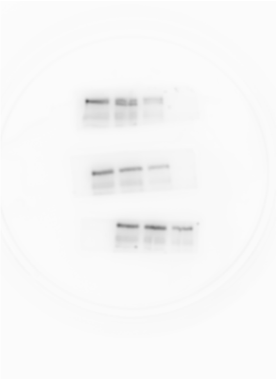

β -actin(1)

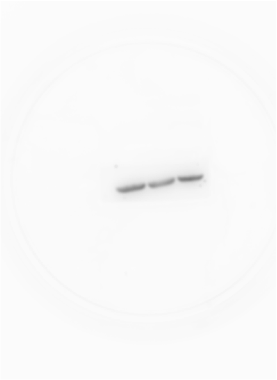

β -actin(2)

β -actin(3) and (4):
the second and third brands

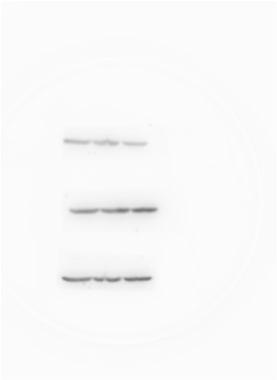

Figure2b:

ATF4 (1)

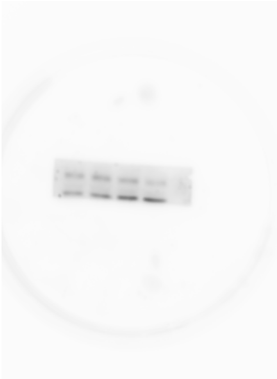

ATF4 (2)

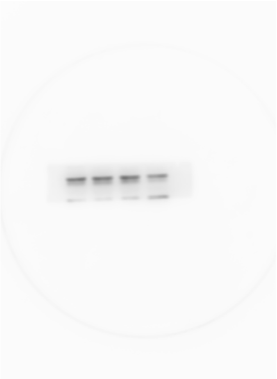

ATF4 (3)

ATF4 (4)

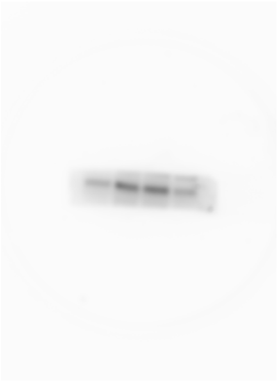

SIAH2(1)

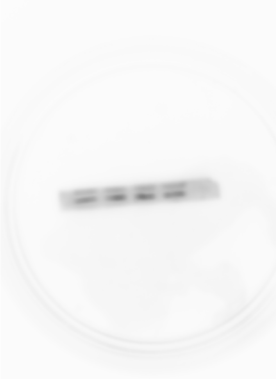

SIAH2(2)

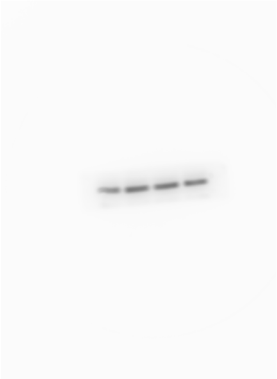

SIAH2(3)

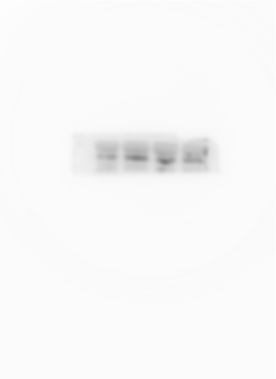

SIAH2(4)

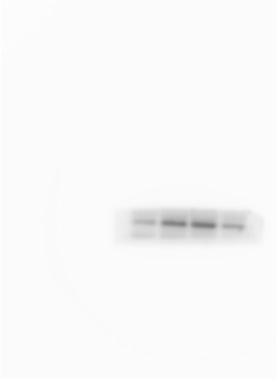

HIPK2(1):
the second brand

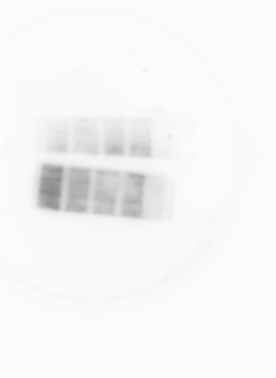

HIPK2(2)

HIPK2(3):
the first brand

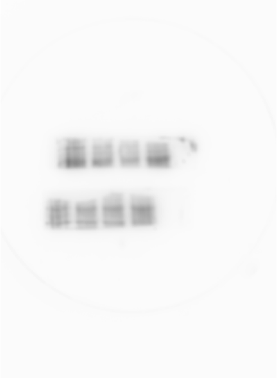

HIPK2(4)

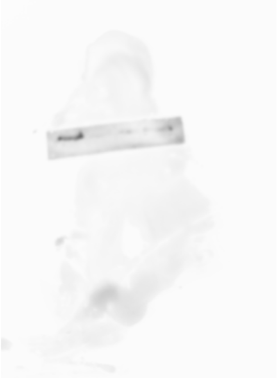

β -actin(1) and (2)

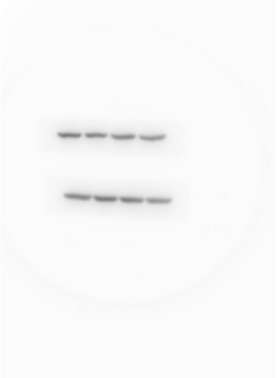

β -actin(3)

β -actin(4)

Figure2c:

ATF4 (1)

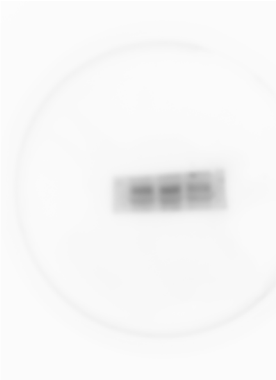

ATF4 (2)

ATF4 (3)

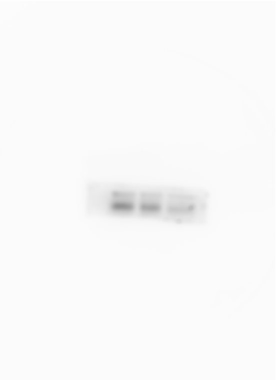

ATF4 (4)

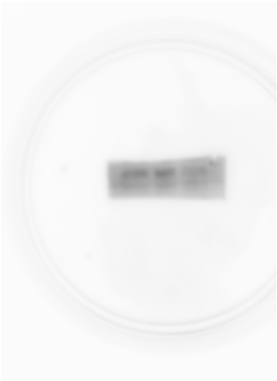

β -actin(1)

β -actin(2)

β -actin(3):
the first brand

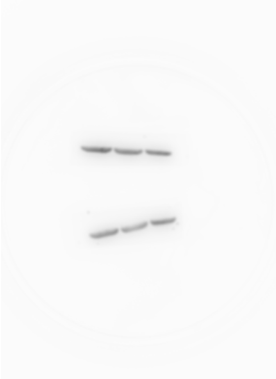

β -actin(4)

Figure2d:

SIAH2(1)

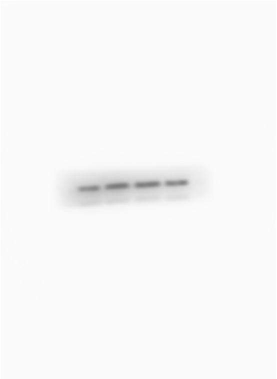

SIAH2(2)

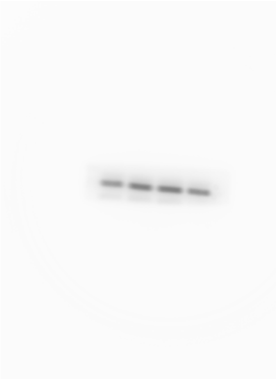

SIAH2(3)

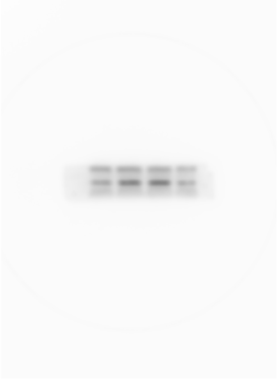

SIAH2(4)

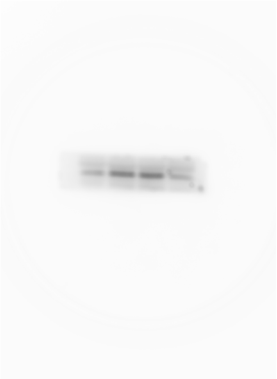

HIPK2(1)

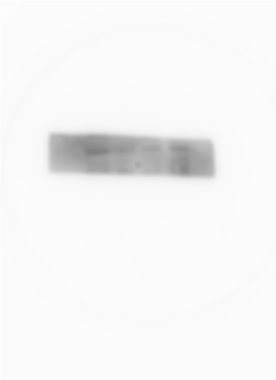

HIPK2(2)

HIPK2(3)

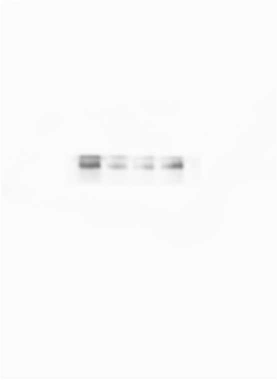

HIPK2(4)

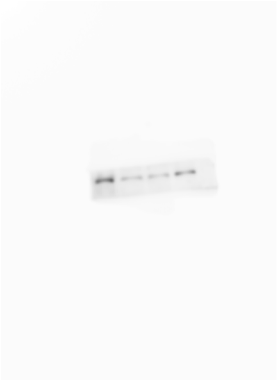

β -actin(1)

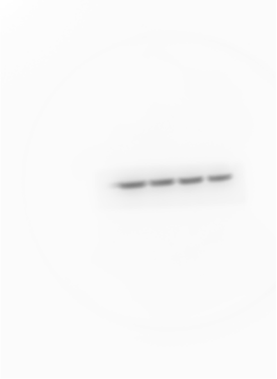

β -actin(2)

β -actin(3)

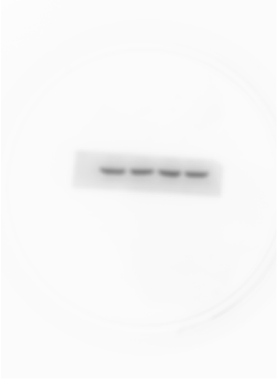

β -actin(4)

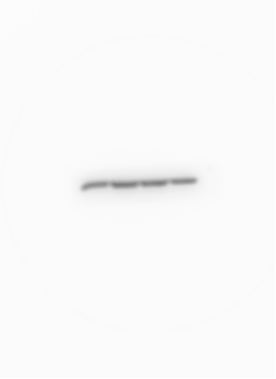

Figure2e:

SIAH2(1)

SIAH2(2)

SIAH2(3)

SIAH2(4)

β -actin(1)

β -actin(2)

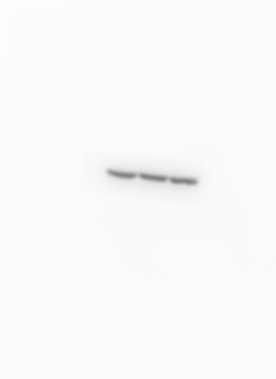

β -actin(3)

β -actin(4)

Figure2f:

HIPK2(1)

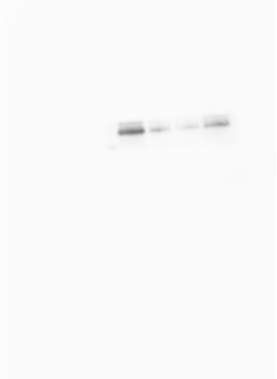

HIPK2(2)

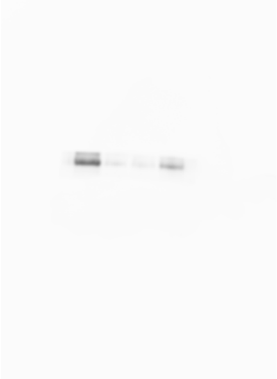

HIPK2(3)

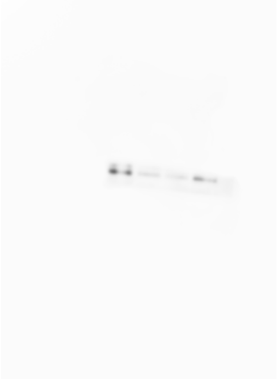

HIPK2(4)

β -actin(1)

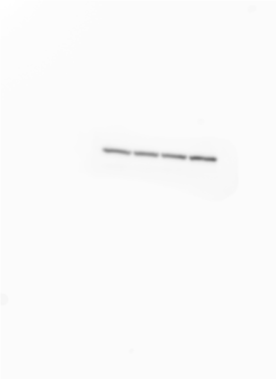

β -actin(2)

β -actin(3)

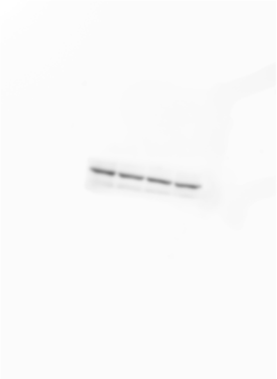

β -actin(4)

Figure4:

PERK(1)

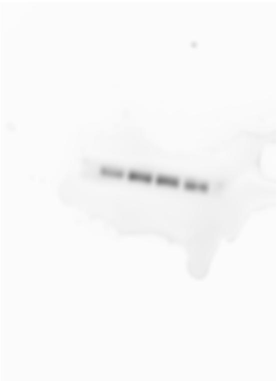

PERK(2)

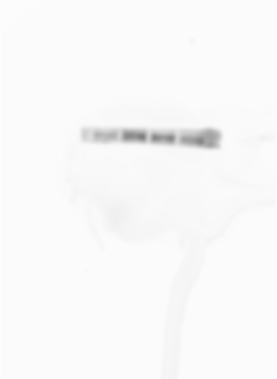

PERK(3)

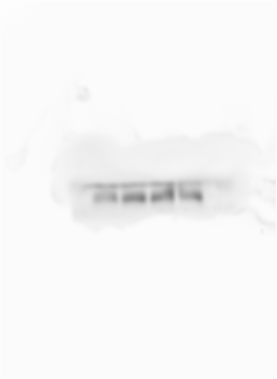

PERK(4)

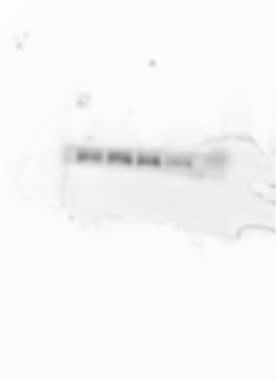

ATF4 (1)

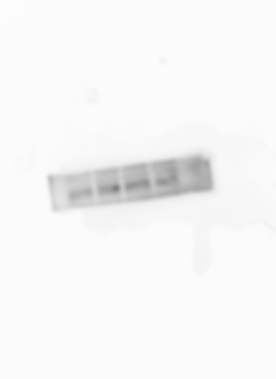

ATF4 (2)

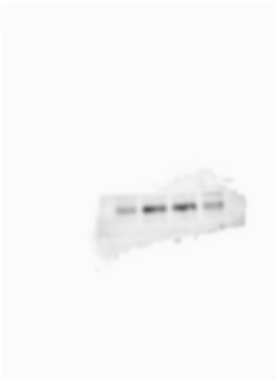

ATF4 (3)

ATF4 (4)

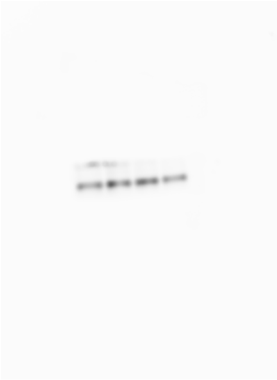

SIAH2(1)

SIAH2(2)

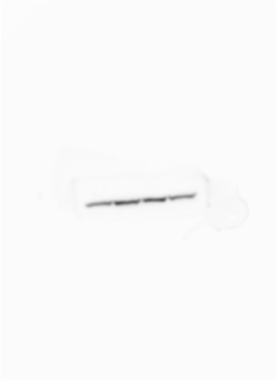

SIAH2(3)

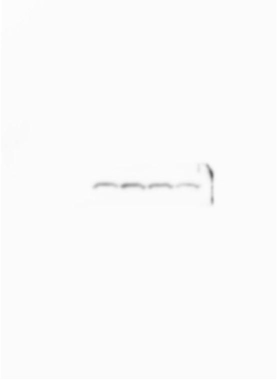

SIAH2(4)

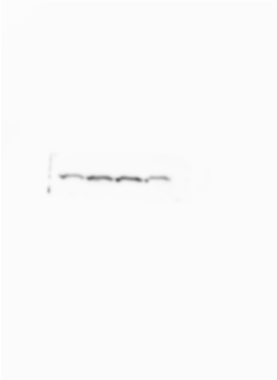

HIPK2(1)

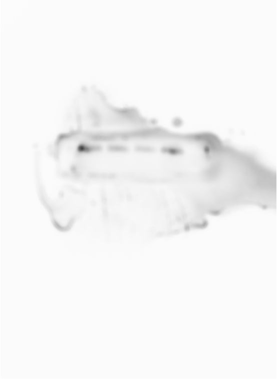

HIPK2(2)

HIPK2(3):

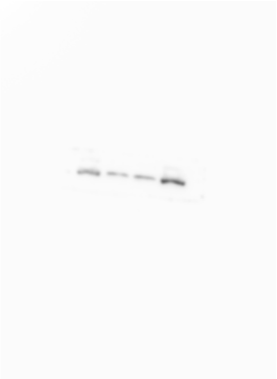

HIPK2(4)

β -actin(1)

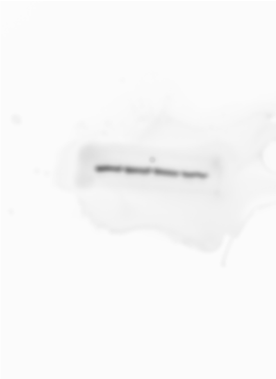

β -actin(2)

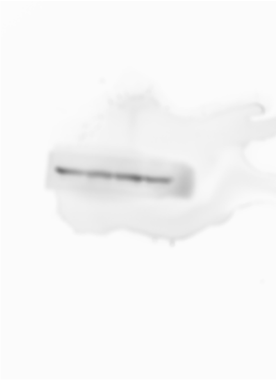

β -actin(3)

β -actin(4)

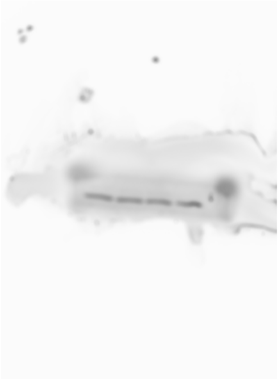

Figure5:

PERK(1)

PERK(2)

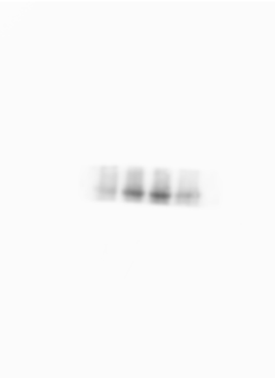

PERK(3)

PERK(4)

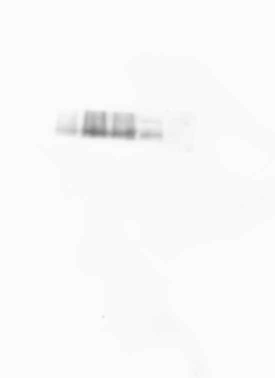

ATF4 (1)

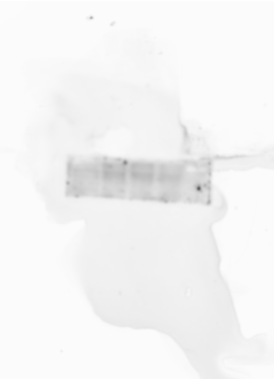

ATF4 (2)

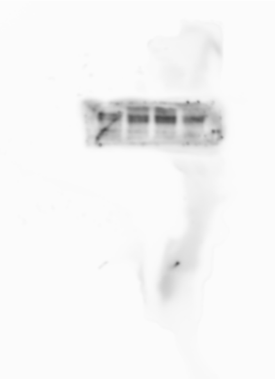

ATF4 (3)

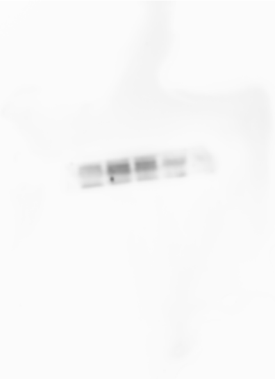

ATF4 (4)

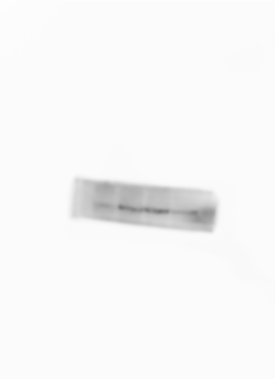

SIAH2(1)

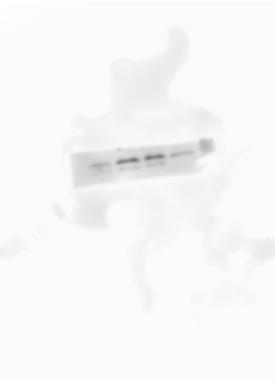

SIAH2(2)

SIAH2(3)

SIAH2(4)

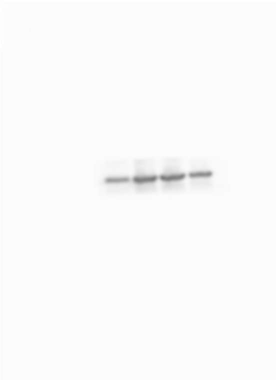

HIPK2(1)

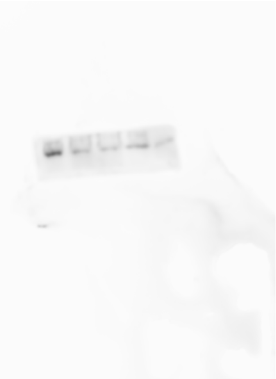

HIPK2(2)

HIPK2(3): the right lane

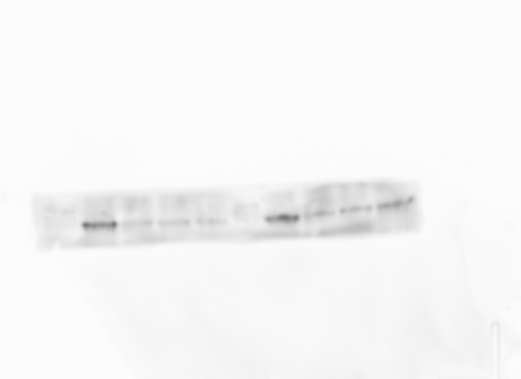

HIPK2(4)

β -actin(1)

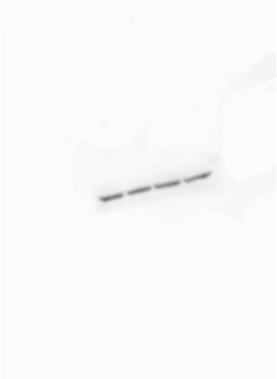

β -actin(2)

β -actin(3)

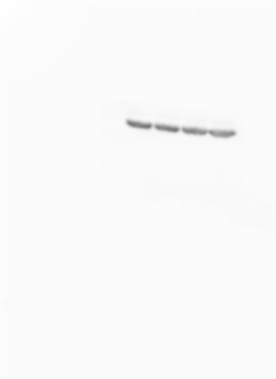

β -actin(4)

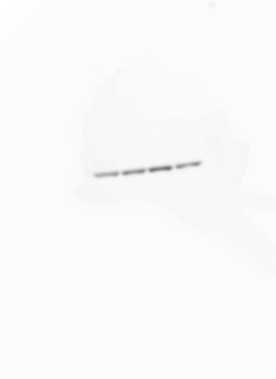

Figure6a:

PERK(1)

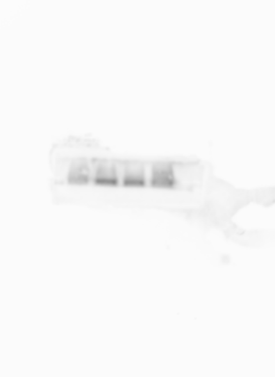

PERK(2)

PERK(3)

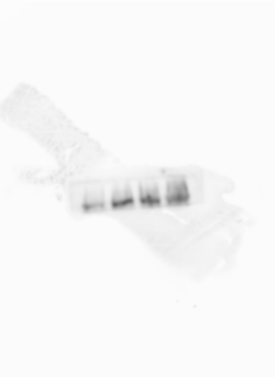

PERK(4)

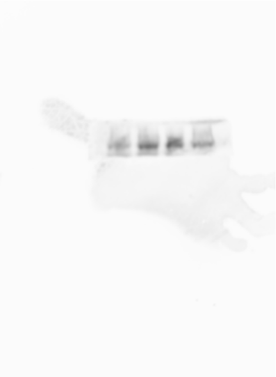

ATF4 (1)

ATF4 (2)

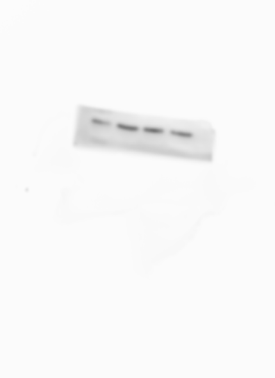

ATF4 (3)

ATF4 (4)

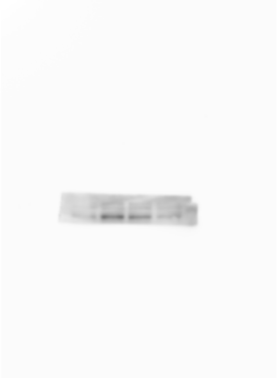

SIAH2(1)

SIAH2(2)

SIAH2(3)

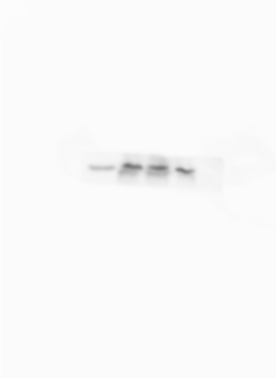

SIAH2(4)

HIPK2(1)

HIPK2(2)

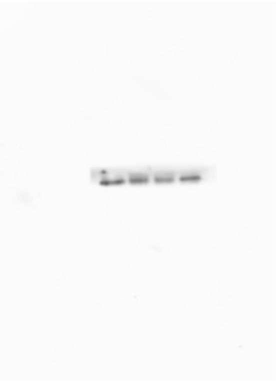

HIPK2(3)

HIPK2(4)

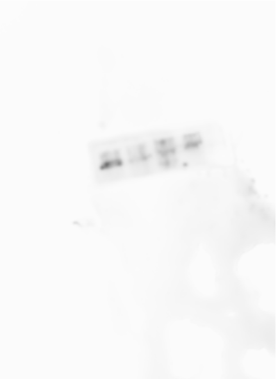

β -actin(1)

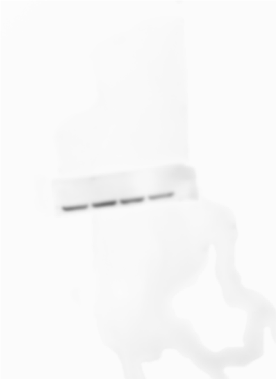

β -actin(2)

β -actin(3)

β -actin(4)

Figure6d:

SIAH2(1)

SIAH2(2)

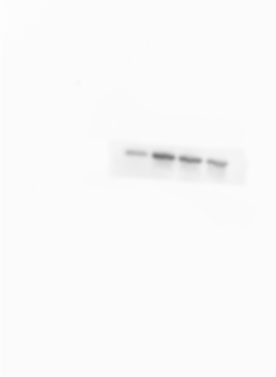

SIAH2(3)

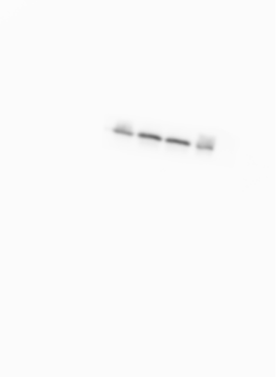

SIAH2(4)

HIPK2(1)

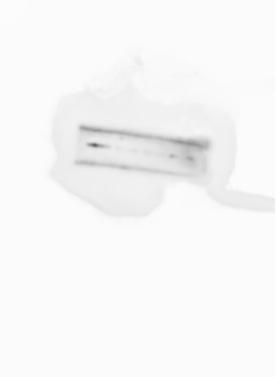

HIPK2(2)

HIPK2(3)

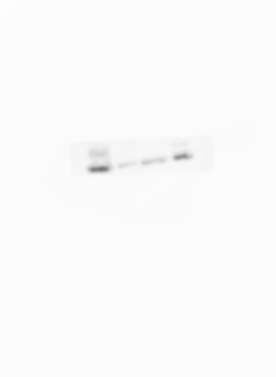

HIPK2(4)

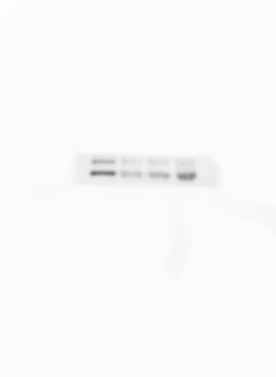

β -actin(1)

β -actin(2)

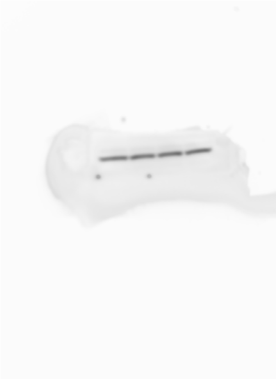

β -actin(3)

β -actin(4)

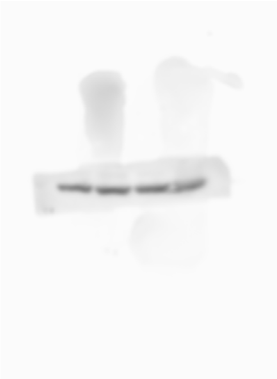

Figure7:

PERK(1)

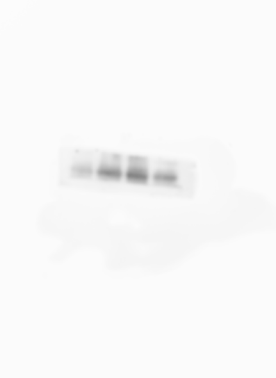

PERK(2)

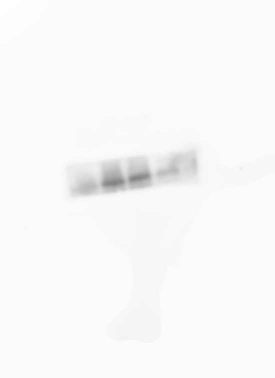

PERK(3)

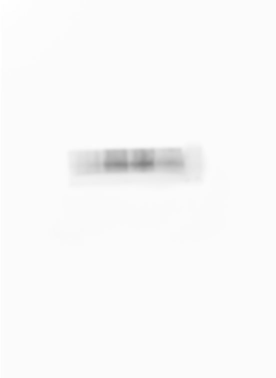

PERK(4)

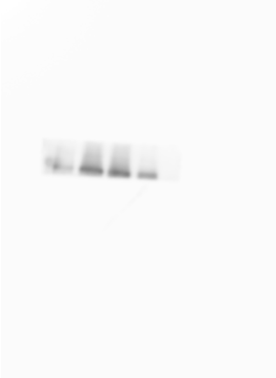

ATF4 (1)

ATF4 (2)

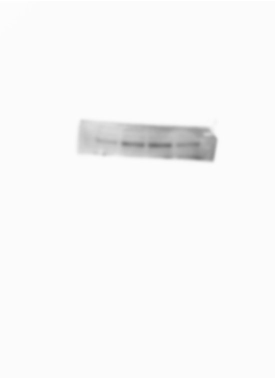

ATF4 (3)

ATF4 (4)

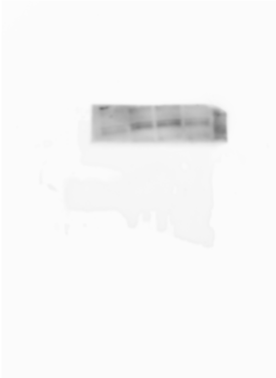

SIAH2(1)

SIAH2(2)

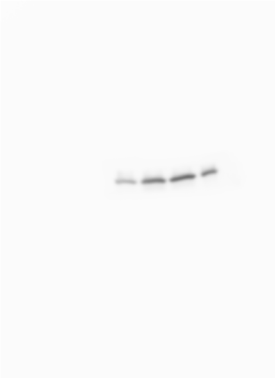

SIAH2(3)

SIAH2(4)

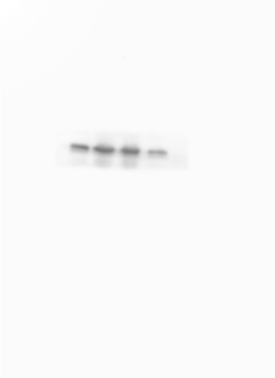

HIPK2(1)

HIPK2(2)

HIPK2(3)

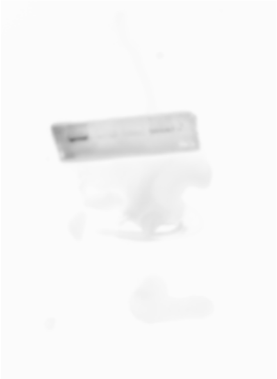

HIPK2(4)

β -actin(1)

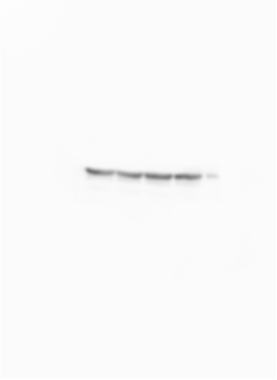

β -actin(2)

β -actin(3)

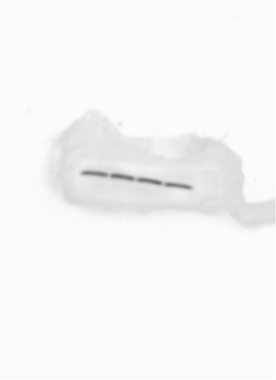

β -actin(4)

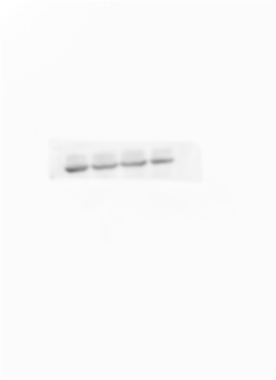

Supplement: Supplementary file 1 — Additional file 1. The original blot images for Figs. 1, 2, 4, 5, 6 and 7. Expression of PERK, ATF4, SIAH2 and HIPK2 were determined from each group using immunoblotting. [file 12931_2023_2454_MOESM1_ESM.pdf]
